# Independent effect of gestational weight gain and prepregnancy obesity on pregnancy outcomes among Saudi women: A sub-cohort analysis from Riyadh mother and baby cohort study (RAHMA)

**DOI:** 10.1371/journal.pone.0262437

**Published:** 2022-01-11

**Authors:** Amel Fayed, Hayfaa A. Wahabi, Samia Esmaeil, Roaa Elkouny, Hala Elmorshedy, Hanadi Bakhsh

**Affiliations:** 1 Clinical Sciences Department, College of Medicine, Princess Nourah Bint Abdulrahman University, Riyadh, Saudi Arabia; 2 Research Chair of Evidence-Based Healthcare and Knowledge Translation, King Saud University, Riyadh, Saudi Arabia; 3 Department of Family and Community Medicine, King Saud University Medical City and College of Medicine, Riyadh, Saudi Arabia; 4 College of Medicine, AlFaisal University, Riyadh, Saudi Arabia; Waikato Institute of Technology, NEW ZEALAND

## Abstract

**Background:**

Gestational weight gain (GWG) and prepregnancy obesity are garnering more attention as determining factors of pregnancy outcomes when it comes to the wellbeing of both the mother and her baby. This study was conducted to describe the pattern of GWG among participants of Riyadh Mother and Baby Multicenter Cohort Study (RAHMA) and to investigate the detrimental effects of excessive GWG and prepregnancy obesity on pregnancy outcomes.

**Methods:**

RAHMA is a multicentre cohort study conducted in three hospitals in Riyadh, Saudi Arabia. Participants were categorized according to the Institute of Medicine into inadequate, adequate, and excessive GWG, and stratified by body mass index (BMI) into under/normal weight, overweight, and obese. To examine the independent effect of maternal prepregnancy obesity and GWG, a multivariate regression model was used and adjusted odds ratio (AOR) and 95% Confidence Interval (CI) for each outcome were calculated.

**Results:**

A total of 7029 participants were included in this study; 31.8% had adequate GWG, 25.9% had excessive GWG and 42.3% had inadequate GWG, while 29.7% had normal BMI, 33.3% were overweight, 34.8% were obese, and 2.2% were underweight. Excessive GWG was independently associated with increased risk of hypertensive events, (AOR = 1.77, 95% CI 1.20–2.63). Obesity was associated with higher risk of gestational diabetes (AOR 2.11, 95% CI 1.76–2.53), hypertensive events (AOR 2.06, 95% CI 1.48–3.01), and delivery by emergency caesarean section (AOR = 1.63, 95% CI 1.35–1.97). Infants of obese women had increased odds of macrosomia (AOR 3.11, 95% CI 1.94–4.99) and lower odds of low birth weight (AOR = 0.68, 95% CI 0.53–0.88).

**Conclusion:**

In comparison to excessive GWG, which increases the risk of hypertensive events during pregnancy, prepregnancy obesity is associated with more adverse outcomes including GDM, hypertensive events in pregnancy and emergency CS.

## Introduction

Gestational weight gain (GWG) and prepregnancy obesity are garnering more attention as determining factors of pregnancy outcomes when it comes to the wellbeing of both the mother and her baby [1–3]. GWG is the amount of maternal weight gained between conception and the time of birth to accommodate the growing fetus. In 1990, the Institute of Medicine (IOM) of the United States of America released formulated recommendations for healthy GWG during pregnancy [4]. Since that time many aspects of the health of women of childbearing age have changed. In 2009, the IOM modified the 1990 guidelines based on findings from research performed over almost two decades following their initial recommendations. The updated IOM guidelines included recommendations for GWG in twin pregnancy, changes to the recommendations for obese women, and a recommendation that all women should strive to be within the normal body mass index (BMI) range when they conceive [5].

The IOM concluded that prepregnancy body mass index (BMI) was an important predictor of birth weight, independent of maternal GWG, and that prepregnancy BMI should be used to guide recommendations for GWG. Thus, determining BMI became an integral part of the physical examination of pregnant women [3].

Physiologic weight gain during pregnancy can be attributed primarily to the weight of the developing fetus and to increases in maternal body water, blood volume and fat. Physiologic changes related to pregnancy result in optimum weight gain for women with a normal BMI from 11 to 16 kg [6].

In recent years, adverse pregnancy outcomes have been linked to both inadequate and excessive GWG. Excessive GWG (above the IOM target range) is associated with increased risk for postpartum weight retention, obesity, and the related co-morbidities such as chronic hypertension (HTN), pregnancy-related HTN (gestational HTN, preeclampsia), gestational diabetes (GDM) or type 2 diabetes mellitus. In addition to the increased risk of obstetric complications including caesarean section delivery (CS), and the birth of a macrosomic infant [6–9].

GWG during pregnancy is also associated with offspring health, as the risk for childhood obesity increases when GWG is excessive [10].

Nevertheless, insufficient GWG (below the IOM target range) is associated with increased incidence of preterm delivery, birth of a small-for-gestational age infant, and low birthweight (LBW) [6, 9].

Maternal obesity carries an increased risk of many adverse pregnancy outcomes. A recent systematic review of cohort studies showed that almost 24% of pregnancy complications were related to maternal obesity [11]. Such adverse effects included increased rate of caesarean section (CS) delivery, HTN, GDM, large for gestational age infants and stillbirths [12, 13]. Furthermore, infants born to obese mothers are at increased risk of cardiac and metabolic diseases in addition to childhood obesity [14].

There is high prevalence of prepregnancy obesity and overweight among Saudi women with documented association with GDM, induction of labour, pregnancy associated HTN, CS delivery and macrosomia [15–17].

Despite the various pregnancy outcomes being dependent on GWG, it is seldom discussed as an independent entity from prepregnancy obesity. This is particularly true in Saudi Arabia with literature addressing GWG being outdated or including a relatively small sample size [18, 19]. The aim of this study is to describe the pattern of GWG among participants of Riyadh Mother and Baby Multicenter Cohort Study (RAHMA), to predict the associated risk factor, and to analyse the independent impact of excessive GWG and prepregnancy obesity on the incidence of adverse maternal and fetal outcomes.

## Materials and methods

### Study design and participants

This study was approved by the Institutional Review Boards at each participating hospital and followed Helsinki’s declaration guidelines for research on human subjects. King Abdullah International Medical Research Centre, approval letter 11/062; King Fahad Medical City Research Centre, approval letter 013–017; and King Saud University, approval letter 13–985.

Further to Institution Review Approval from all participating hospitals, participating mothers gave written informed consent.

RAHMA is a multicentre cohort study conducted in three hospitals in Riyadh, Saudi Arabia which are King Khalid University Hospital, King Fahad Medical City which is run by the Ministry of Health (MOH), and King Abdul-Aziz Medical City, which is affiliated to the National Guards. Recruitment of the cohort commenced in November 2013 and data collection concluded in March 2015. A stratified cluster random sampling approach was used to choose the participating hospitals. The kind of hospital, which comprised (MOH), military, and university hospitals, was used as a stratifying variable [17].

All mothers who gave birth in one of the participating hospitals, as well as their babies, were eligible to be enrolled in the study. Before signing a written informed consent, the moms were given written information about the study in Arabic. Non-Saudi women were not included in the study [17].

A standardised data collection sheet was used. Data were obtained from both the maternal and infant medical records in addition to an in-person post-delivery maternal self-administrated questionnaire (within 2 days after delivery), which provided data on the mothers’ sociodemographic conditions (**S1 File**). Further details of the study methodology are available in the cohort profile report [17].

In this sub-cohort we excluded pregnancies with multiple gestations and women with missing data on prepregnancy BMI. Comparison of the main outcome determining factors of excluded and included population is provided in **S1 Table**.

The study population were grouped into three groups of GWG (inadequate, adequate and excessive GWG) according to Institute of Medicine (IOM) classification [5], and stratified into three groups according to prepregnancy BMI (under/normal weight, overweight, obesity).

### Outcome variables

- Gestational Diabetes (GDM) and pre-gestational Diabetes based on the cut-off values used by the World Health Organization (WHO) [20].
- Hypertensive disorders of pregnancy based on the report of the American national working group on high blood pressure in pregnancy [21]. As the incidence of pre-eclampsia and eclampsia were low, all hypertensive events were aggregated in one group for analysis.
- Maternal prepregnancy body mass index (BMI) was calculated from weight prior to pregnancy and height measured during the first antenatal clinic. Participants were classified according to the WHO BMI definitions as follows: underweight ≤18.4 kg/m^2^, healthy weight (18.5–24.9 kg/m^2^), overweight (25.0–29.9 kg/m^2^) and obese (≥ 30 kg/m^2^) [22].
- Gestational Weight gain (GWG) was calculated as the difference between the term weight and the prepregnancy weight.
- Classification of GWG was adopted according to Institute of Medicine (IOM) [5]. The IOM guidelines recommend the following cut-off values for adequate GWG:
  ➢ a total weight gain of 12.5–18 Kg for underweight BMI women (≤18.5 kg/m^2^)
  ➢ a total weight gain of 11.5–16 kg for normal BMI women (18.5–24.9 kg/m^2^)
  ➢ a total weight gain of 7–11.5 kg for overweight women (BMI of 25–29.9 kg/m^2^)
  ➢ a total weight gain of 5–9 kg for all obese women (BMI of 30 kg/m^2^or greater)
- Macrosomia is defined as a birth weight at term of ≥ 4.0 kg [23].
- Low birth weight (LBW) is defined as a birth weight < 2.5 kg at term [24].
- Preterm birth is defined as birth that takes place before 37 weeks gestation [25].

### Statistical analysis

Description of all study variables in form of mean ±standard deviation (for quantitative variables) or frequency and percentages (for qualitative variables) was done. Comparison of continuous variables among the three GWG groups was done using Analysis of Variance (ANOVA) after testing of normality distribution. Association between GWG and various categorical variables was tested using Chi-Square tests. For the independent effect of obesity and GWG on the pregnancy outcome, we examined effect modification of GWG categories stratified by prepregnancy BMI categories for each pregnancy outcome using Chi-square and crude Odds Ratio.

Multivariable logistic regression was used to examine the independent association between pregnancy outcomes and GWG. The covariates included in the model were those of proven influence on GWG. The models were adjusted for maternal age, parity and gestational age as continuous variables. GWG and prepregnancy BMI were analysed as categorical variables where adequate GWG was used as the reference group for GWG and normal prepregnancy BMI group was considered as the reference group for prepregnancy obesity.

We tested multiplicative interaction by including cross-product terms for GWG categories and prepregnancy BMI in the multivariable model. Adjusted odds ratio (AOR), with confidence intervals (CIs) at 95% were presented, and statistical significance was set at two tailed with p-value < 0.05. Statistical analyses were performed with IBM SPSS version 21 [26] and Stata 16.0 [27].

## Results

A total of 7029 participants were included in this study. According to the IOM guideline [5], 31.8% had adequate GWG during pregnancy, while 25.9% had excessive GWG and 42.3% had inadequate GWG. Only 157 women (2.2%) were classified as underweight while 2338 (33.3%) were overweight and 2447 (34.8%) were obese.

**Fig 1:**
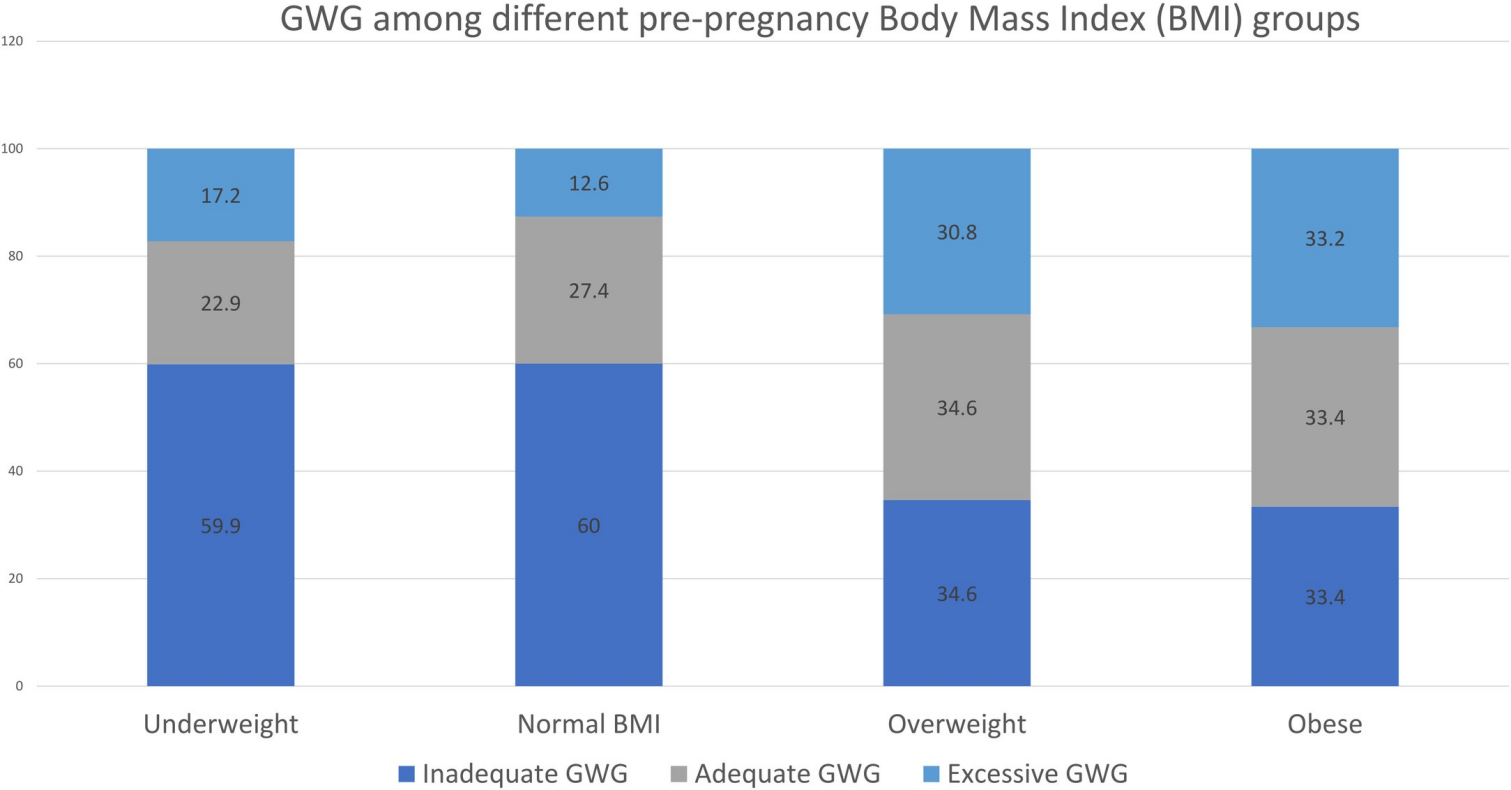
Distribution of gestational weight gain according to institute of medicine among various prepregnancy body mass index groups.

Participants in different IOM groups were of comparable age, exposure to SHS as well as history of pre-gestational diabetes and HTN (Table 1). Women with excessive GWG have significantly higher prepregnancy BMI and gestational age at delivery compared to women with inadequate GWG (P<0.05) (Table 1). Nevertheless, participants with inadequate GWG were of significantly higher parity (Table 1). Inadequate GWG was more common among underweight and normal prepregnancy BMI while excessive GWG was more prevalent among overweight and obese women (Fig 1).

**Table 1:** Participant characteristics by institute of medicine gestational weight gain categories.

Characteristics	Inadequate (N = 2972)	Adequate (N = 2236)	Excessive (N = 1821)	p-value
**Age (years)**	29.7±5.9	29.7±5.9	29.8±5.7	0.75
<20	78(2.6)	49(2.2)	40(2.2)	0.69
20–29	1432(48.2)	1126(50.4)	898(49.3)	
30–34	789(26.5)	587(26.3)	476(26.1)	
35+	673(22.6)	474(21.2)	407(22.4)	
**Prepregnancy BMI (kg/m** ^**2**^ **)**	27.1±5.8	28.6±5.7	29.4±5.4	<0.001
Underweight/normal BMI	1346 (45.3)	607 (27.2)	289 (15.9)	<0.01
Overweight	808 (27.2)	810 (36.2)	720 (39.5)	
Obese	817 (27.5)	818 (36.6)	812 (44.6)	
**Parity**	2.46±2.22	2.31±2.14	2.11±2.04	
0	630(21.2)	520(23.3)	475(26.1)	0.003
1–4	1461(49.2)	1061(47.5)	852(46.8)	
5+	881(29.6)	654(29.3)	492(27.0)	
**Second-hand smoker**	615(24.9)	482(26.6)	357(24.2)	0.24
**Pregestational Diabetes**	78(3.7)	55(3.6)	64(5.1)	0.09
**Pregestational hypertension**	34(1.1)	24(1.1)	32(1.8)	0.102
**Gestational age at delivery (weeks)**	38.7±2.0	38.9±1.9	38.9±1.7	<0.001

Data are expressed in frequency (percentage) or mean± Standard deviation, BMI = Body Mass Index.

**Fig 2:**
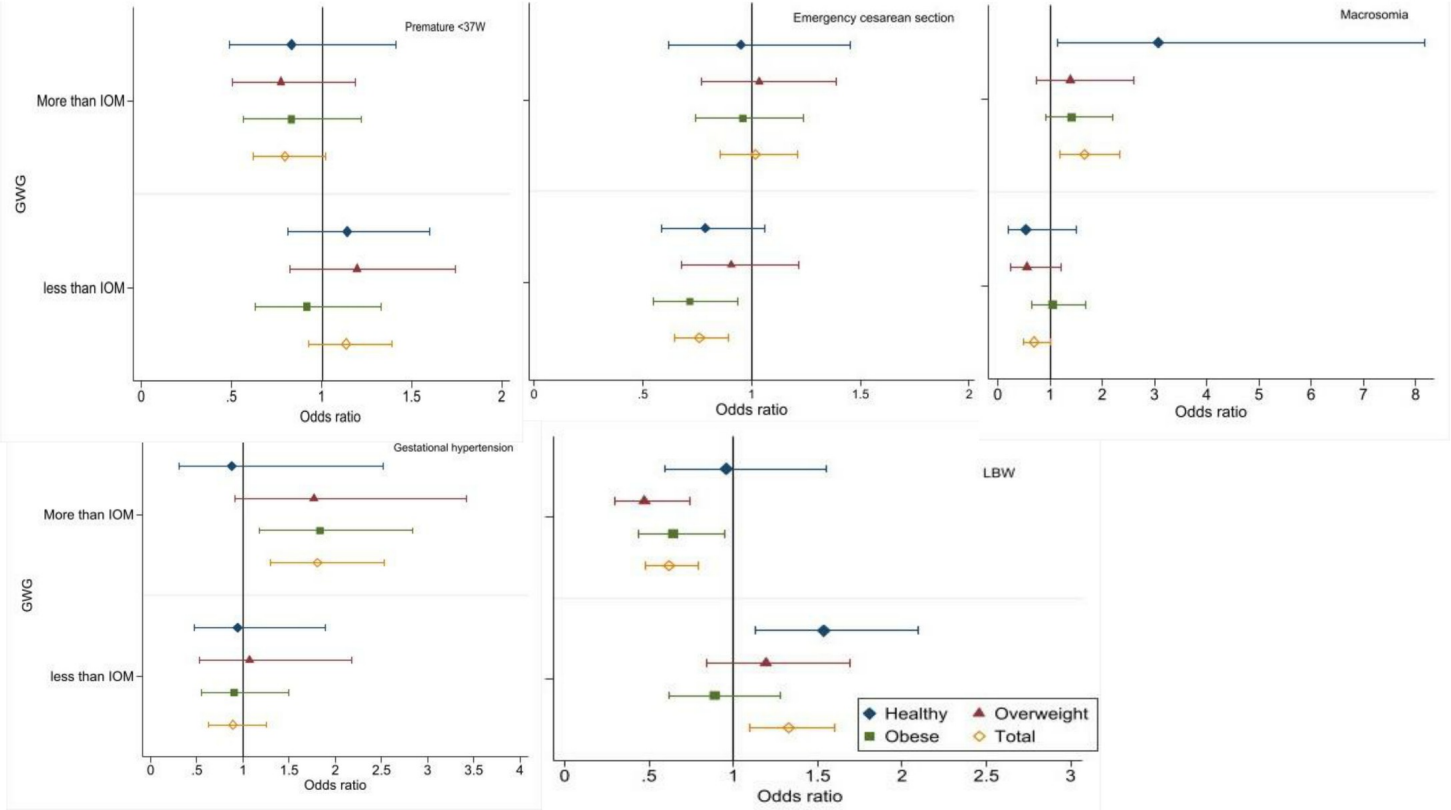
Association of pregnancy outcomes with gestational weight gain among participants according to the prepregnancy body mass index.

Compared to women with adequate GWG, women who gained excess weight had significantly higher probability of developing HTN during pregnancy (4.8% versus 2.7%), deliver a macrosomic baby (4.9% versus 3.0%), and they were less likely to have an LBW baby (5.4% versus 8.5%) (Table 2). Additionally, they had a non-significant greater odds of emergency CS delivery and GDM. Women who gained inadequate weight were less likely to deliver by CS (11.6% versus 14.8%) and more probably to have LBW (11.0% versus 8.5%) (Fig 2 and Table 2).

**Table 2:** Association of pregnancy outcomes with gestational weight gain among participants according to the prepregnancy body mass index.

Outcome	Inadequate (N = 2972)	Adequate (N = 2236)	Excessive (N = 1821)	P-value
BMI category	Event/Total (%)	Event/Total (%)	Event/Total (%)	
**Emergency Caesarean section**				
All women	346 (11.6)	330 (14.8)	273(15.0)	<0.001
Underweight/normal BMI	138 (10.2)	77 (12.7)	35 (12.1)	0.25
Overweight	98 (12.1)	107 (13.2)	98 (13.6)	0.665
Obese	110 (13.5)	146 (17.8)	140 (17.2)	<0.001
**HTN**				
All women	71(2.4)	60(2.7)	86 (4.8)	<0.001
Underweight/normal BMI	25 (1.9)	12 (2.0)	5 (1.7)	0.969
Overweight	16 (2.0)	15 (1.9)	23 (3.2)	0.150
Obese	30 (3.7)	33 (4.1)	58 (7.2)	0.002
**Gestational Diabetes**				
All women	497 (24.6)	371 (25.0)	324 (27.4)	0.183
Underweight/normal BMI	161 (18.0)	70 (17.3)	34 (16.7)	0.891
Overweight	125 (22.3)	105 (20.3)	104 (24.2)	0.357
Obese	211 (37.1)	196 (34.8)	186 (33.9)	0.509
**Preterm birth**				
All women	254 (8.7)	169 (7.8)	111(6.3)	0.010
Underweight/normal BMI	132 (10.0)	52 (8.8)	21 (7.5)	0.67
Overweight	64 (8.1)	54 (6.8)	38 (5.4)	0.119
Obese	58 (7.3)	63 (7.9)	52 (6.6)	0.644
**Macrosomia**				
All women	55 (2.1)	61 (3.0)	84 (4.9)	<0.001
Underweight/normal BMI	8 (0.7)	7 (1.3)	10 (3.8)	0.002
Overweight	10 (1.4)	18 (2.4)	23 (3.3)	0.045
Obese	38 (5.0)	36 (4.8)	51 (6.6)	0.225
**Low Birth Weight**				
All women	326 (11.0)	190 (8.5)	99 (5.4)	<0.01
Underweight/normal BMI	191 (14.2)	59 (9.7)	27 (9.3)	0.005
Overweight	75 (9.3)	64 (7.9)	28 (3.9)	<0.001
Obese	60 (7.3)	67 (8.2)	44 (5.4)	0.079

Data are expressed in frequency (percentage) or mean± Standard deviation, BMI = Body Mass Index.

Women with obesity were less likely to deliver by CS if they restricted their GWG (13.5% versus 17.2%). Compared to women with normal BMI, obese women, had less probability to deliver an LBW baby; (5.4% versus 9.3%) and nearly six-times the possibility to develop HTN during pregnancy; (7.2% versus 1.7%) if they had excessive GWG (Fig 2 and Table 2).

The effect of GWG on the different pregnancy outcomes was attenuated after adjusting of all confounders and its significant effect maintained only for CS, HTN and birth weight, whilst the independent effect of prepregnancy obesity was significantly associated with all pregnancy outcomes except the preterm birth (Table 3). Excessive GWG significantly doubled the HTN risk (AOR = 1.77, 95% CI 1.20–2.63) and diminished the risk of LBW by 39% (AOR = 0.61, 95% CI 0.41–0.90) (Table 3). Restricting the GWG significantly lowered the risk of CS by 30% (AOR = 0.70, CI 0.54–0.90) and the risk of macrosomia by 61% (AOR = 0.39, 95% CI, 0.20–0.76). Compared to women with normal prepregnancy BMI, women with prepregnancy obesity had an increase of the risk of emergency CS; (AOR = 1.63, 95% CI 1.35–1.97,), nearly double the risk of HTN (AOR = 2.06, 95% CI 1.48–3.03) and GDM (AOR = 2.11, 95% CI 1.76–2.53), and increased risk of delivering a macrosomic baby by 1.59 times (95% CI 1.11–2.27), meanwhile, they had lower risk of LBW by 32% (AOR = 0.68, 95% CI 0.53–0.88) (Table 3).

**Table 3:** Independent effect of gestational wight gain and prepregnancy body mass index on different pregnancy outcomes.

Pregnancy outcome	Inadequate GWG	Excessive GWG	Prepregnancy Obesity
	AOR (95% CI)	AOR (95% CI)	AOR (95% CI)
**Emergency Caesarean section¶**	0.70 (0.54–0.90) *	1.07 (0.85–1.12)	1.63 (1.35–1.97) *
**Any hypertensive event in pregnancy§**	0.93(0.59–1.47)	1.77 (1.20–2.63) *	2.06 (1.48–3.03) *
**Gestational Diabetes§**	1.06 (0.87–1.31)	1.10 (0.90–1.35)	2.11 (1.76–2.53) *
**Preterm birth^**	1.16 (0.90–1.48)	0.83 (0.63–1.05)	0.90 (0.67–1.22)
**Macrosomia¶**	0.39 (0.20–0.76) *	1.07 (0.65–1.76)	3.11 (1.94–4.99) *
**Low Birth Weight¶**	1.31 (0.95–1.81)	0.61 (0.41–0.90) *	0.68 (0.53–0.88) *

Abbreviations: GWG; gestational Weight Gain, AOR; adjusted odds ratio, CI; confidence interval, BMI; Body Mass Index.

Inadequate GWG and Excessive GWG are compared to women within IOM GWG recommendation.

Prepregnancy Obesity is compared to women with normal prepregnancy Body Mass Index.

**¶**Adjustment of maternal age, parity, Gestational Diabetes, Gestational Hypertension, gestational age at delivery.

§ adjusted for of maternal age, parity, gestational age at delivery.

^ Adjustment of maternal age, parity, Gestational Diabetes, Gestational Hypertension.

*p-value less than 0.05.

Testing the interaction between GWG and prepregnancy BMI was not statistically significant when considered in the regression models.

## Discussion

The results of this study showed that a larger proportion of obese and overweight women had excessive GWG compared to women with normal prepregnancy weight or those who were underweight. The study showed that maternal obesity was associated with GDM, hypertensive events in pregnancy and emergency CS, while excessive GWG was associated only with hypertensive events in pregnancy. Neonates of obese mothers in this sub-cohort were more likely to be macrosomic and less likely to be of LBW. Conversely, women with inadequate GWG, irrespective of their BMI, were less likely to deliver by emergency CS or to deliver a macrosomic baby. This study showed no association between prepregnancy weight or GWG and preterm birth among Saudi women.

The greater influence of prepregnancy obesity on the adverse outcome of pregnancy compared to excessive GWG was documented by previous studies, however obese women with excessive GWG are at the greatest risk of adverse pregnancy outcomes [11, 28, 29].

Previous studies showed that obesity is associated with hypertensive disorders of pregnancy and GDM which is in line with our results [30, 31]. Published reports showed that obesity increases the risk of pre-eclampsia by 3 to10-fold compared to normal weight women with an additional 1.4 to 5-fold increase in the risk of venous thromboembolism in pregnancy and the postpartum period [32, 33]. Moreover, obesity is reported to increase the risk of GDM by 4–9 times compared to women with normal prepregnancy weight which agrees with findings in this study [34]. It has been suggested that in countries with a high prevalence of maternal obesity, such as in the case of Saudi Arabia, control of obesity prior to pregnancy may result in 14–35% fewer women with GDM and hypertensive disorders in pregnancy [35]. Thus, control of obesity among Saudi women in the reproductive age group will have a major impact on maternal and child health considering the high prevalence of GDM of 24% which we have observed and reported previously [17]. In this study, excessive GWG was associated with hypertensive disorders in pregnancy. Previous studies reported conflicting results for the effect of excess GWG on hypertensive events in pregnancy with some studies suggesting the association [36, 37] and other studies rejecting such association [28, 38]. However, a recently published study confirmed the association between excessive body fluid which was associated with excessive GWG as the main factor responsible for preeclampsia [39].

It is not surprising that obese women in this study had almost twofold the risk of CS delivery compared to normal weight women considering the high prevalence of obstetric complications such as preeclampsia and GDM in addition to the increased risk of fetal macrosomia among them. Similar findings were reported in other cohorts [40, 41]. The risk of CS delivery was found to increase by 1.4-fold in overweight women compared to normal weight women and almost three times more in morbidly obese women [41]. This observation suggests a linear relationship between the risk of CS delivery and maternal obesity. We did not observe an association between excessive GWG and CS delivery in this cohort, albeit, such association was recently reported in a systematic review of 23 observational studies [8].

High birthweight is more common in pregnancies complicated by maternal obesity than in pregnancies of normal weight women [34]. Our results showed that obesity increased the risk of macrosomia by almost threefold. The birth of a big baby is associated with multiple maternal and neonatal complications including postpartum haemorrhage, birth canal injuries, shoulder dystocia and birth fracture [42].

Contrary to the findings of other investigators [8], we did not find an association between inadequate GWG and LBW or PTB, however, we have documented the association of prepregnancy underweight and LBW in the same cohort in a recent publication [15].

Our findings on the effects of low GWG in decreasing the odds for macrosomia and CS delivery have practical application in women with prepregnancy overweight and obesity in reducing the rate of these adverse outcomes. Similar findings were reported by other investigators [43, 44] with the recommendations of tailoring weight gain for individual woman based on the degree of obesity and the balance between avoidance of adverse effects of excessive weight gain and inadequate weight gain [45, 46].

It is noteworthy that differences in the prevalence of obesity, overweight, and excessive weight gain were observed between different ethnic groups [47, 48], independent of socioeconomic and lifestyle characteristics. This observation supports further investigation into the optimum range of GWG in Saudi women with a high BMI to achieve the least pregnancy adverse outcomes, guided by the recommendation of the IOM.

We found conflicting reports about the effects of weight loss in obese women during pregnancy. Some reports showed a significant reduction in the adverse outcomes associated with weight loss with no significant increase in the rate of PTB, or LBW, while other reported an increased rate of small for gestational age infants [49, 50].

In this study, prepregnancy obesity was observed to have a greater effect on adverse pregnancy outcomes than excessive GWG, which may be explained by the effects of the maternal insulin resistance, hyperinsulinemia and inflammation mediators, which seem to contribute to placental and fetal dysfunction in early pregnancy before the effect of GWG is established [51]. This premise is further supported by the limited success of different lifestyle and dietary interventions during pregnancy in improving fetal overgrowth, preeclampsia or GDM, which are adverse effects of prepregnancy obesity rather than excessive GWG [52–54].

### Implications of the study findings to practice and research

- Effective interventions including various modalities of diet and exercise should be employed to reduce weight gain during pregnancy and prepregnancy obesity as well as weight retention during the postpartum period [55, 56].
- Targeting Saudi women in the reproductive age group with health education about the possible serious adverse effects of obesity on their reproductive life. Such health education should be part of the school and university curricula as over 90% of the women are attending schools or universities.
- Further research should be directed to the investigation of community specific interventions to reduce the burden of obesity among reproductive age group women and to the investigation of effects of maternal obesity on Intrauterine programming and the neonate, child, and adult future health [57].

### Strength and limitations

This is the first study from Saudi Arabia to investigate the effect of prepregnancy weight and GWG on different maternal and perinatal outcomes. The large cohort of over 7000 participants included in the study provided an accurate and specific account of the effect of prepregnancy weight and GWG on the outcome of pregnancy among Saudi mothers with consideration of all covariates in the analysis. The study will offer important results for health services planning for intervention to reduce prepregnancy obesity and overweight as well as for targeting high risk pregnancy in women with high BMI with specific healthcare. We are aware of the limitation of this study including the observation nature of the investigation and the lack of data on some outcomes such as postpartum weight retention (PPWR), nevertheless, we have investigated PPWR in a follow-up study of the same cohort [58]. In addition, we had to exclude a considerable number of the participants due to incomplete data on the main determinants of the investigation, however, we find no significant differences between the population included in this study and the one excluded (**S1 File**).

## Conclusion

In comparison to excessive GWG, which increases the risk of hypertensive events during pregnancy, prepregnancy obesity is associated with more adverse outcomes including GDM, hypertensive events in pregnancy and emergency CS.

## Supporting information

S1 File Data collection sheet. (XLSX)

S1 Table Comparison of included and excluded participants from the originally recruited cohort. (DOCX)
